# Comparison of Power Ultrasound and NALC-NaOH Decontamination Methods for Stool Mycobacterial Culture: A Prospective Study

**DOI:** 10.3390/microorganisms12091799

**Published:** 2024-08-30

**Authors:** Peng Tian, Jing He, Xiaojie Ling, Yan Wang, Yunfeng Deng, Zhongfa Zhang

**Affiliations:** 1 Katharine Hsu International Research Center of Human Infectious Diseases, Shandong Public Health Clinical Center, Shandong University, Jinan 250013, China; jazz198611@163.com (P.T.); yanerw@126.com (Y.W.); 2 Shandong Provincial Clinical Research Center for Infectious Diseases, Shandong Public Health Clinical Center, Shandong University, Jinan 250013, China; hejing17393147859@163.com

**Keywords:** tuberculosis, decontamination methods, NALC-NaOH, power ultrasound, stool

## Abstract

Stool samples have been reported to be useful for the diagnosis of pulmonary tuberculosis (PTB), especially in patients who are unable to produce sputum. However, contamination limits the usefulness of stool specimens in mycobacterial culture. In this study, a novel decontamination method of power ultrasound (PU) was evaluated for mycobacterial isolation from suspected PTB cases. Stool samples (*n* = 650) were collected, and each sample was divided into approximately three equal groups. In addition to an AFB smear (Auramine O method), the stool samples were treated using different decontamination methods (NaOH-NALC vs. PU methods). The sensitivity (calculated against CRS) and contamination rates between the two methods were compared using McNemar’s test. Of the 650 samples, 32 (4.92%) stool samples treated with the NaOH-NALC method were culture-positive, including *Mycobacterium tuberculosis* (M.TB; *n* = 21, 3.23%) and nontuberculous mycobacteria (NTM; *n* = 11, 1.69%). Sixty-one (9.38%) stool samples treated with the PU method were culture-positive, including M.TB (*n* = 37, 5.69%) and NTM (*n* = 24, 3.69%). Statistical analysis showed that a significant difference was found in the isolation rate of M.TB and NTM between the two methods (*p* < 0.05). Additionally, compared with the NALC-NaOH method (19.07%), stool samples treated with the PU method (13.23%) had a significantly lower contamination rate (*p* < 0.05). In conclusion, our findings suggest that the utilization of the PU method as a novel decontamination technique could significantly enhance the isolation rates of both NTM and M.TB when stool specimens are employed for culture. Compared to the NaOH-NALC method, this approach proves to be more effective in facilitating stool mycobacterial culture.

## 1. Introduction

Tuberculosis (TB) remains a significant global public health challenge. According to the Global Tuberculosis Report 2023, it caused an estimated 1.3 million deaths in 2022, with approximately 410,000 individuals worldwide affected by multidrug-resistant/rifampicin-resistant tuberculosis (MDR/ RR-TB). However, the treatment success rate for drug-resistant tuberculosis (DR-TB) stands at a mere 63% [1]. The emergence of DR-TB significantly impedes efforts towards TB prevention and treatment. The timely and accurate diagnosis of TB or DR-TB is essential for reducing its prevalence and transmission. The World Health Organization (WHO) recommends that a TB diagnosis should be based on acid-fast bacilli (AFB) smear microscopy, rapid molecular methods, or sputum mycobacterial culture [2]. While rapid molecular methods, such as Xpert, provide prompt results including rifampin resistance and are increasingly being globally adopted for the diagnosis of pulmonary tuberculosis (PTB), sputum culture remains the gold standard for PTB diagnosis. Current diagnostic algorithms still necessitate the isolation of cultured strains for subsequent drug susceptibility testing (DST) to isoniazid and other medications [3].

In clinical practice, sputum is the predominant sample used for the diagnosis of PTB. However, in certain patient populations, such as young children, the elderly, severely ill individuals, those living with human immunodeficiency virus (HIV), or pregnant women, sputum is not always available [3,4,5,6,7,8,9]. Therefore, alternative specimens such as induced sputum, bronchoalveolar lavage fluid, or gastric lavage fluid are required. But these specimens have many disadvantages, such as being invasive and costly as well as requiring special equipment that may not be accessible in resource-constrained settings.

Because sputum is regularly swallowed, *Mycobacterium tuberculosis* (M.TB) can be detected in stool by mycobacterial culture [10]. Stool samples can be collected conveniently and demonstrate diagnostic potential for PTB. However, intestinal flora can result in bacterial contamination and lead to the diminished sensitivity of the culture method. Therefore, prior to mycobacterial culture, it is necessary to perform digestion and decontamination of stool specimens. The most commonly employed approach for this purpose is the N-acetyl-L-cysteine (NALC)-sodium hydroxide (NaOH) method [11]. Despite its widespread use in stool decontamination, the NALC-NaOH method may not be the optimal procedure due to several limitations. For instance, although NaOH is considered a decontamination agent, it also exhibits toxicity towards mycobacterial isolates [12], potentially leading to false negative results in paucibacillary infection cases such as those observed in patients or children with HIV [13]. In addition, NALC reagents should be freshly prepared (valid for less than one day), further limiting the practicality and utility of the NALC-NaOH method. In previous years, various methods have been attempted to improve the decontamination of stool specimens for the isolation of *Mycobacterium*. However, these methods are either limited in their applicability or lack field evaluations [14,15,16,17,18]. Consequently, there is a need for the development of a novel decontamination procedure specifically tailored to stool specimens.

Recently, power ultrasound (PU, 20 to 100 kHz) has been introduced into biomedical research [19], primarily due to its bactericidal properties [20]. Moreover, the susceptibility to PU depends on the species of pathogens, indicating its pathogen-specific nature [21,22,23]. Previously, PU technology was found, during sputum mycobacterial culture, to effectively eliminate various fast-growing microbiological agents and enhance the isolation rate of M.TB [24]. In this study, we applied PU technology to stool mycobacterial culture for decontamination and assessed its value. Additionally, we compared its performance with that of the NALC-NaOH decontamination method.

## 2. Materials and Methods

### 2.1. Subjects

The present study was conducted at the Katharine Hsu International Research Center of Human Infectious Diseases, Shandong Public Health Clinical Center, Shandong University. Between April and September 2023, consecutively enrolled patients with suspected PTB were prospectively assessed prior to the administration of anti-TB therapy. The attending clinicians used composite reference standards (CRSs) to diagnose PTB [25], extrapulmonary tuberculosis (EPTB) [25], and nontuberculous mycobacterial (NTM) disease [26] among the enrolled patients. The CRSs define a fixed, transparent rule to classify subjects into disease-positive and disease-negative groups based on existing imperfect tests [27].

### 2.2. Stool Samples

Stool samples were collected following the guidelines of the “Tuberculosis Laboratory Test Protocol (2015)” [28]. Subsequently, each stool sample was divided into approximately three equal groups. In addition to performing direct AFB smear microscopy (Baso, Zhuhai, China) using the Auramine O method, the stool samples were processed using different decontamination methods (NaOH-NALC vs. PU methods). Mycobacterial culture was performed using MGIT 960 (Becton Dickinson, Franklin Lakes, NJ, USA). Isolates were identified by MALDI-TOF MS (Bruker Daltonics, Bremen, Germany) or 16S rRNA gene sequencing.

### 2.3. Direct AFB Smear Microscopy (Auramine O Method)

A 0.1 g stool sample was suspended in 6 mL of sterile 0.067 M phosphate-buffered saline (PBS, pH 6.8) and homogenized by vortexing. Subsequently, the mixture was allowed to settle for a duration of 10 min [28]. A single droplet of the resulting supernatant was carefully placed onto a microscope slide and subjected to air drying, followed by staining using the Auramine O method. The slides were examined using a light-emitting diode fluorescent microscope (magnification ×400) (Shunyu iLED, Ningbo, China) independently by two experienced laboratory technicians. However, to measure inter-reader variability, the technicians were blinded to each other’s smear interpretations.

### 2.4. Digestion and Decontamination Methods (DDMs)

NaOH-NALC method [28]: A 0.1 g stool sample was suspended in 6 mL of sterile PBS and homogenized by vortexing. The mixture was then allowed to settle for 10 min. Subsequently, 2 mL of the supernatant was mixed with 2 mL of NaOH-NALC solution (composed of 4% NaOH, 2.9% sodium citrate, and 0.5% NALC, resulting in a final NaOH concentration of 1%). This mixture was incubated at room temperature for 15 min. Following incubation, 40 mL of sterile PBS was added, and the solution was centrifuged at 3000× *g* for 15 min. The supernatant was discarded, and the sediment was resuspended in 200 μL of sterile PBS and homogenized for 5 s with a vortex mixer (Scilogex, Hillsborough Township, NJ, USA). Finally, 500 μL of the resuspension was used for MGIT culture, and one drop was used for AFB smear microscopy (Auramine O method).

**Figure 1:**
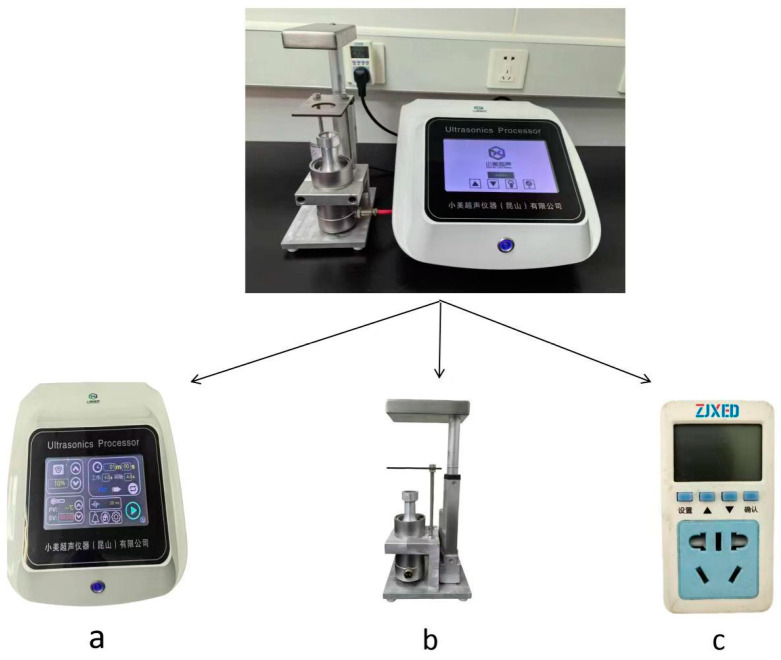
The non-contact power ultrasound meter. Parameters for ultrasound: frequency (20 kHz). The power density (D, W/mL) of the ultrasound dissipated into the medium with volume V is given by D = P/V, where P is the input power. The power intensity (I, W/cm^2^) dissipated from a probe tip with radius r is given by I = P/(πr^2^). The symbol “πr^2^” represents the cross-sectional area of the ultrasonic probe, where π is approximately equal to 3.14. (**a**) Microprocessor controller; (**b**) ultrasonic transducer; (**c**) input power monitor.

**Figure 2:**
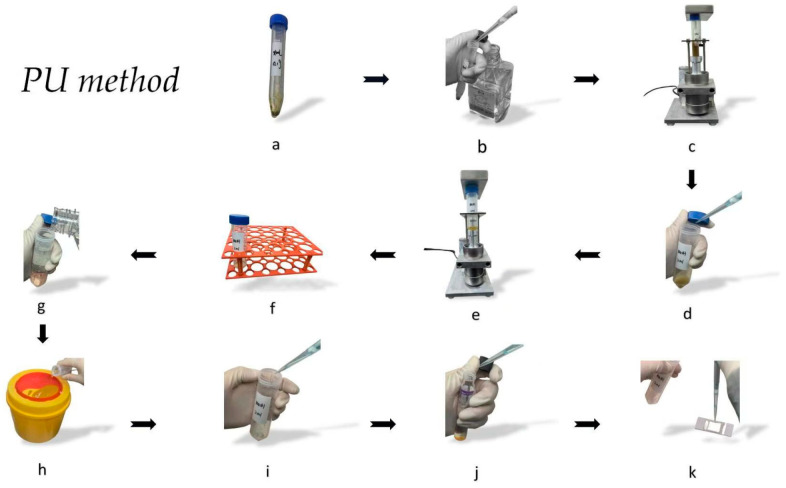
The PU method procedure. (**a**) Measured 0.1 g of the stool sample; (**b**) mixed with 6 mL of PBS; (**c**) underwent treatment with PU meter; (**d**) settled for 10 min, and 2 mL of the supernatant was mixed with an equal volume of 2% NaOH; (**e**) treated in the PU meter again; (**f**) incubated at room temperature for 10 min; (**g**) added 40 mL of PBS; (**h**) centrifuged, discarded the supernatant; (**i**) resuspended in 200 μL sterile PBS; (**j**) MGIT culture; (**k**) AFB smear microscopy. All the photographs were taken directly by our team using a HUAWEI Mate 60 Pro (HUAWEI, Shenzhen, China).

PU method: A 0.1 g stool sample was mixed with 6 mL of sterile PBS in a 15 mL BD Falcon centrifuge tube. The centrifuge tube was then secured onto a specialized tube rack and placed inside the non-contact PU meter (Figure 1), where it underwent treatment with an input power of 100 W (ultrasonic frequency, 20 kHz; power density, 20 W/mL; power intensity, 7.96 W/cm^2^) and sonication for 1 min. Immediately after sonication, the tube was removed from the PU meter (Xiaomeichaosheng, Kunshan, China) and allowed to settle for 10 min. After settling, 2 mL of the supernatant was mixed with an equal volume of 2% NaOH (resulting in a final concentration of 1% NaOH) in a 50 mL BD Falcon centrifuge tube. This mixture was then treated in the PU meter again (input power, 19 W; ultrasonic frequency, 20 kHz; power density, 4.75 W/mL; power intensity, 1.51 W/cm^2^; time, 1 min), followed by incubation at room temperature for 10 min. Subsequently, 40 mL of PBS was added, and the sample was centrifuged at 3000× *g* for 15 min. The rest of the procedure was as described in the NaOH-NALC method (Figure 2).

### 2.5. Statistical Analysis

Statistical analysis was performed using SPSS (version 26.0, IBM, Armonk, NY, USA). Categorical variables are described using frequency (percentage) and compared utilizing the chi-square test, while continuous variables are presented as median (P25, P75) and were compared using the Wilcoxon signed-rank test. Sensitivity was calculated against CRSs, and the contamination rate of each method is reported. Sensitivity and contamination rates between the two methods were compared using the McNemar test. Agreement between the NaOH-NALC and PU methods was calculated using the kappa value. A *p*-value of less than 0.05 was considered significant.

## 3. Results

### 3.1. Baseline Characteristics

**Figure 3:**
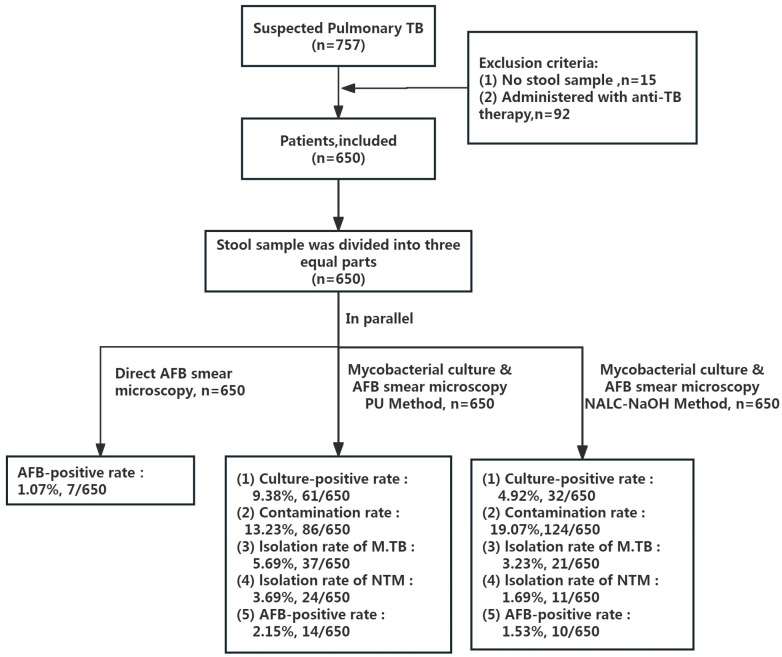
Patient selection.

A total of 650 patients (age: 52.18 ± 20.88 years; men: 58.61%, 381/650) were recruited for our study, and the corresponding stool samples were collected. Figure 3 and Table 1 show the recruitment process and characteristics of the cohort. Of the 650 patients, 77 (11.85%) were diagnosed with PTB (including PTB with EPTB, *n* = 28), 27 (4.15%) were diagnosed with NTM diseases, and 546 (84.00%) had an alternative diagnosis.

### 3.2. Sensitivities (NaOH-NALC vs. PU Methods)

Of the 650 samples, 32 (4.92%) stool samples decontaminated with the NaOH-NALC method were culture-positive, including M.TB (*n* = 21, 3.23%) and NTM (*n* = 11, 1.69%). In contrast, 61 (9.38%) stool samples decontaminated with the PU method were culture-positive, including M.TB (*n* = 37, 5.69%) and NTM (*n* = 24, 3.69%). Therefore, the PU method detected 16 additional cases of M.TB and 13 additional cases of NTM compared to the NaOH-NALC method.

In addition, out of the 650 samples, only 7 (1.07%) cases were direct AFB smear microscopy positive. However, AFB smear microscopy detected in 10 (1.53%) of the samples processed by the NALC-NaOH method and 14 (2.15%) of those by the PU method. It was apparent that the PU method treatment, when used for decontamination, detected 4 and 7 additional positive cases compared to the NALC-NaOH method and direct AFB smear microscopy, respectively. Nevertheless, all four additional positive cases decontaminated using the PU method exhibited positive results in culture but yielded negative culture outcomes when processed using the NaOH-NALC method (Table 2).

The AFB smear microscopy and culture results of patients with suspected pulmonary TB are presented in Table 3. Statistical analysis showed a significant difference in the culture-positive frequency of M.TB and NTM between the two method groups (*p* < 0.05). The agreement between the NaOH-NALC and PU methods was fairly good (kappa = 0.784, *p* < 0.001). For M.TB detection, the PU method achieved a higher sensitivity compared to that of the NaOH-NALC method (McNemar test, *p* < 0.0001).

### 3.3. Contamination Rate (NaOH-NALC vs. PU Methods)

Of the 650 stool samples, 124 (19.07%) were considered contaminated when decontaminated with the NALC-NaOH method, and 86 (13.23%) were considered contaminated when using the PU method (Table 3). A significant difference between them was observed (*p* < 0.05).

### 3.4. Time to Positive (TTP, NaOH-NALC vs. PU Methods)

Among the 650 stool samples, 32 (4.92%) stool samples were culture-positive when subjected to decontamination using both methods, including M.TB (*n* = 21, 3.23%) and NTM (*n* = 11, 1.69%). The median TTP for M.TB (*n* = 21) detection using the PU method was 9.83 days (7.25, 12.96), which significantly outperformed the NALC-NaOH method (10.87 days (7.93, 14.39)). This disparity in detection time exhibited statistical significance (*Z* = −3.78, *p* < 0.001). Similarly, the median TTP to detect NTM (*n* = 11) by the PU method was found to be 8.08 days (5.33, 11.71), compared to the median detection time (8.71 days (6.67, 11.83)] of the NALC-NaOH method, and this difference in TTP was significant (*Z* = −2.936, *p* = 0.003) (Table 4).

## 4. Discussion

TB remains one of the top 10 causes of mortality worldwide and was the number one cause of death attributed to a single infectious disease prior to the COVID-19 pandemic, surpassing HIV/AIDS in ranking [1]. Timely identification of TB plays a pivotal role in ensuring optimal patient care, treatment, and effective TB control. As the gold standard, mycobacterial culture remains an efficient tool for the diagnosis of active TB [2,3].

Sputum is the primary specimen used in diagnosing PTB. In practice, obtaining sputum samples from patients with suspected PTB is not always feasible. This poses a challenge in TB diagnosis, often necessitating invasive procedures like bronchoscopy and lung puncture. Recently, stool samples have emerged as valuable alternative specimens for diagnosing childhood PTB and HIV complicated with PTB [3,4,5,6,7]. However, stool specimens in culture suffer from significant contamination issues, limiting their diagnostic utility in PTB [3]. In our study, we discovered that the PU method, as a novel decontamination technique, could enhance the isolation rate of both NTM and M.TB when stool specimens were utilized, compared to the NaOH-NALC method. Additionally, we observed a lower contamination rate in stool samples treated with the PU method compared to those treated with the NaOH-NALC method. These intriguing findings suggest that the PU method surpasses the NaOH-NALC method in the decontamination process, potentially improving the diagnostic yield of mycobacterial culture.

The achievement of satisfactory culture results can be enhanced by the utilization of efficient DDMs. However, the NALC-NaOH method, which is currently the most widely used DDM in stool mycobacterial culture, still encounters several limitations. For example, although 1% NaOH is considered a decontamination agent, it is also toxic to mycobacterial isolates [12,13]. In addition, NALC should be prepared every day, further limiting the practicality and utility of the NALC-NaOH method. Currently, limited progress has been made in the decontamination procedure for stool samples. In this study, the PU technique was successfully introduced to treat stool for mycobacterial culture. Our data suggest that, compared with the NALC-NaOH method, more M.TB isolates were isolated from stool treated using the PU method. Compared with the NALC-NaOH method, the PU method has several advantages. First, it could reduce the decontamination time, from 15 min to 10 min. Second, without freshly prepared NALC, the PU method is easier to use and more cost-effective. Third, before digestion and decontamination, the stool samples underwent ultrasonic dispersion at 100 W to enhance the dispersal of Mycobacteria present in the feces within the samples, thus facilitating their isolation. Fourth, time-to- positive (TTP), which reflects the metabolic activity of M.TB in liquid medium and has a negative correlation with the activity of inoculated bacteria, exhibited a relatively low level in the group treated with the PU method [14]. Fifth, comparing the cost of the two methods, the approximate cost (excluding instrument cost) for the PU method and the NALC-NaOH method was found to be USD 8.6627 and 8.6679, respectively. These findings support that the PU method is superior to the NALC-NaOH method in the isolation of M.TB from stool samples.

Furthermore, the PU method facilitated the detection of additional NTM cases. With the rising incidence of NTM diseases [15], the PU method holds significant potential for widespread use. This advantage of the PU method may be attributed to differences in the response of NTM strains to various DDMs. However, further investigation is warranted. NTM is widely distributed in the environment, making contamination unavoidable. Therefore, in this study, NTM culture was repeated in 24 patients, and the data supported our finding that NTM isolation was not solely due to contamination.

Contamination poses a significant challenge for timely diagnosis in stool mycobacterial culture [29]. Improving DDMs may reduce stool culture contamination, allowing for more reliable estimates of its diagnostic utility [3]. In recent decades, numerous DDMs have been evaluated for their effectiveness in reducing stool culture contamination within laboratory settings. The effectiveness of different decontaminating agents in the recovery of M.TB and decontamination was found to be superior with NaOH compared to sulfuric acid and benzalkonium chloride-1-hexadecylpyridinium chloride [30,31]. In general, despite the development of various DDMs for stool treatment, limited progress has been made in this field. Our data indicate that stool treated with the PU method (13.23%) exhibited a significantly lower contamination rate compared to that treated with the NALC-NaOH method (19.07%). Generally, the contamination rate of stool samples treated with NALC and NaOH ranges from 14% to 41% [3,4,5,6,7,8,9,10]. The PU method not only has a shorter decontamination time but also demonstrates superior performance in contamination rates compared to the NALC-NaOH method.

Although sputum AFB smear microscopy has traditionally served as the primary method for the early diagnosis of PTB, its limited sensitivity often leads to diagnostic delays [9]. However, compared to sputum smear microscopy, stool-based direct AFB smear microscopy demonstrates relatively lower sensitivity [32]. In this study, only seven cases (1.07%) showed positive results in direct AFB smear microscopy. After performing centrifugation to collect bacteria for the two methods (NaOH-NALC or PU methods), the positive rate of stool smear exhibited an increase. The PU method detected four additional positive cases compared to the NALC-NaOH method. Nevertheless, all four additional positive cases decontaminated using the PU method showed positive results in culture but yielded negative culture outcomes when processed using the NaOH-NALC method (Table 2). The observed smear results may be attributed to the predigestion and decontamination process of the PU method, which involved subjecting stool samples to ultrasonic dispersion at 100 W. This step aimed to enhance the dispersal of the Mycobacteria present in the stool within the samples, thereby facilitating their isolation. The intriguing findings suggest the potential of the PU method in enhancing the detection rate of stool smears, warranting further investigation.

While our study yielded interesting findings, caution is required due to several limitations. First, the PU parameters were initially designed based on our previous study conducted on sputum samples; these parameters, which were applied to stool samples, may not be the optimal choice for isolating M.TB and NTM from stool samples. Therefore, further optimization is necessary in this regard. Second, additional large-scale and rigorous studies are required to validate our findings thoroughly.

## 5. Conclusions

In conclusion, our findings suggest that the utilization of the PU method as a novel decontamination technique could significantly enhance the isolation rates of both NTM and M.TB when stool specimens are employed for culture. Compared to the NaOH-NALC method, this approach proves to be more effective in facilitating stool mycobacterial culture. However, the precise mechanism underlying its efficacy remains unclear and warrants further investigation.

## Figures and Tables

**Table 1:** Baseline characteristics of included patients.

Variables	Total (*n*)	Pulmonary TB (*n*)
Number	650	77 *
Age, years	52.18 ± 20.88	49.16 ± 19.95
Male, %	58.61	59.74
HIV status (+)	21	17
Extrapulmonary TB		
Pleural	15	15
Lymph node	7	7
Bone joint	7	1
Intestinal	5	5
Clinical manifestations		
Fever	650	77
Cough more than 2 weeks or longer	650	77
Dyspnea	37	24
Hemoptysis	8	8
Microbiological evidence (+)		
AFB smear microscopy	67	34
Rapid molecular methods (PCR and Xpert)	100	70
Culture	102	76
Radiological findings		
X-ray	22	12
CT	600	71
Histopathological evidence		
Pulmonary biopsy	197	70
Extrapulmonary biopsy	60	28

* The diagnosis of 77 cases of PTB was established using a composite reference standard (CRS), with microbiological confirmation obtained in 76 cases, while a single case relied exclusively on clinico-pathological evidence for diagnosis. TB, tuberculosis; HIV, human immunodeficiency virus; AFB, acid-fast bacilli; PCR, polymerase chain reaction.

**Table 2:** A comparative analysis was conducted on the results of AFB smear and culture obtained from different DDMs using the same stool samples.

			Culture					
			PU Method (*n*)			NaOH-NALC Method (*n*)		
			P.	N.	C.	P.	N.	C.
Direct AFB smear microscopy		P.	7	0	0	7	0	0
		N.	54	503	86	25	494	124
AFB smear microscopy	PU method (*n*)	P.	13	0	1	8	4	2
		N.	48	503	85	24	491	122
	NaOH-NALC method (*n*)	P.	9	0	1	8	0	2
		N.	52	503	85	24	494	122

P., positive; N., negative; C., contamination.

**Table 3:** The results of AFB smear microscopy and culture of patients with suspected pulmonary TB, classified by NaOH-NALC and PU methods.

		Total	Pulmonary TB (*n*)	Sensitivity * (%, 95% Confidence Interval)			Contamination Rate (%)
				Mycobacteria **	NTM	M.TB	
Culture	NaOH-NALC method	32	21	30.77 (22.29–40.69)	40.74 (23.01–60.99)	27.27 (18.03–38.81)	19.07
	PU method	61	37	58.65 (48.57–68.09)	88.89 (69.70–97.09)	48.05 (18.03–38.81)	13.23
AFB smear microscopy	NaOH-NALC method	10	5	9.62 (4.96–19.09)	18.52 (7.03–38.75)	6.49 (2.41–15.15)	
	PU method	14	7	13.46 (7.82–21.89)	25.92 (11.87–46.59)	9.09 (4.04–18)	

* Sensitivity (%) was calculated against the composite reference standard (CRS). For the detection of Mycobacteria, sensitivity was calculated against the total number of CRS-diagnosed cases of PTB and NTM disease; for the detection of M.TB, it relied on the total number of CRS-diagnosed cases of PTB; and for detecting NTM, it was based on the total number of CRS-diagnosed cases of NTM disease. ** including NTM and M.TB. TB, tuberculosis; PU, power ultrasound.

**Table 4:** A comparative analysis was conducted on the results of culture and time-to- positive culture obtained from different DDMs using the same stool samples.

		NaOH-NALC Method (*n*)				TTP (Median, PU vs. NaOH-NALC Methods)			
		M.TB	NTM	N.	C.	M.TB (*n* = 21)	*Z* * (P)	NTM (*n* = 11)	*Z* ** (P)
PU method (*n*)	M.TB	21	0	10	6	9.83 days vs. 10.87 days	*Z* = −3.78 (*p* < 0.001)	8.08 days vs. 8.71 days	*Z* = −2.936 (*p* = 0.003)
	NTM	0	11	7	6				
	N.	0	0	477	26				
	C.	0	0	0	86				

* Comparison of the median TTP of M.TB obtained after treatment with PU vs. NaOH-NALC methods. ** Comparison of the median TTP of NTM obtained after treatment with PU vs. NaOH-NALC methods. N., negative; C., contamination; TTP, time-to- positive culture.

## Data Availability

Data are contained within the article.
